# Stunting, adiposity, and the individual-level “dual burden” among urban lowland and rural highland peruvian children

**DOI:** 10.1002/ajhb.22551

**Published:** 2014-04-07

**Authors:** Emma Pomeroy, Jay T Stock, Sanja Stanojevic, J Jaime Miranda, Tim J Cole, Jonathan CK Wells

**Affiliations:** 1 Newnham College, University of CambridgeCambridge, United Kingdom; 2 Division of Biological Anthropology, Department of Archaeology and Anthropology, University of CambridgeCambridge, United Kingdom; 3 Division of Respiratory Medicine, The Hospital for Sick ChildrenToronto, Ontario, Canada; 4 CRONICAS Centre of Excellence in Chronic Diseases and Department of Medicine, School of Medicine, Universidad Peruana Cayetano HerediaLima, Peru; 5 Centre for Paediatric Epidemiology and Biostatistics, Institute of Child Health, University College LondonLondon, United Kingdom; 6 Childhood Nutrition Research Centre, Institute of Child Health, University College LondonLondon, United Kingdom

## Abstract

**Background:**

The causes of the “dual burden” of stunting and obesity remain unclear, and its existence at the individual level varies between populations. We investigate whether the individual dual burden differentially affects low socioeconomic status Peruvian children from contrasting environments (urban lowlands and rural highlands), and whether tibia length can discount the possible autocorrelation between adiposity proxies and height due to height measurement error.

**Methods:**

Stature, tibia length, weight, and waist circumference were measured in children aged 3–8.5 years (*n* = 201). Height and body mass index (BMI) *z* scores were calculated using international reference data. Age-sex-specific centile curves were also calculated for height, BMI, and tibia length. Adiposity proxies (BMI *z* score, waist circumference-height ratio (WCHtR)) were regressed on height and also on tibia length *z* scores.

**Results:**

Regression model interaction terms between site (highland vs. lowland) and height indicate that relationships between adiposity and linear growth measures differed significantly between samples (*P* < 0.001). Height was positively associated with BMI among urban lowland children, and more weakly with WCHtR. Among rural highland children, height was negatively associated with WCHtR but unrelated to BMI. Similar results using tibia length rather than stature indicate that stature measurement error was not a major concern.

**Conclusions:**

Lowland and rural highland children differ in their patterns of stunting, BMI, and WCHtR. These contrasts likely reflect environmental differences and overall environmental stress exposure. Tibia length or knee height can be used to assess the influence of measurement error in height on the relationship between stature and BMI or WCHtR.

Amongst the myriad factors that contribute to obesity risk, a number of studies have reported an association between stunting and excess adiposity (Varela-Silva et al., 2012). This so-called “dual burden” of malnutrition is particularly relevant in low-middle income countries (LMICs: Black et al., 2013; Popkin et al., 1996; Varela-Silva et al., 2012; Victora et al., 2008), where low birth weight and poor growth often exist alongside a transition to more sedentary lifestyles and westernized diets. The mechanisms underlying this association between stunting and obesity and indeed the extent to which the dual burden exists at the individual level remain contentious but are important for devising strategies to reduce the health and economic burdens of obesity.

Stunting and overweight might coexist within individuals because overweight can develop rapidly, whereas the resolution of height deficits from chronic malnutrition may take several generations (Wells and Stock, 2011). Alternatively, there is some evidence that stunted children have an altered body composition and fat distribution (Hoffman et al., 2007; Martins et al., 2004; Mukuddem-Petersen and Kruger, 2004; Wilson et al., 2012) that predisposes them to excess adiposity and abdominal fat distribution. This may be due to greater insulin sensitivity (Martins and Sawaya, 2006) and/or reduced fat oxidation (Hoffman et al., 2000; Leonard et al., 2009) among stunted individuals (although see Said-Mohamed et al., 2012; Wren et al., 1997).

Rapid postnatal growth is also associated with greater adiposity (Chomtho et al., 2008; Dulloo et al., 2006; Howe et al., 2010; Ibáñez et al., 2006; Modi et al., 2006; Monasta et al., 2010; Ong and Loos, 2006; Victora et al., 2007; Wells et al., 2007), so under some circumstances taller children, who have undergone the most rapid postnatal growth, may be at greater risk of obesity (Wells and Cole, 2011). Rapid postnatal growth may be a “catch-up” response to prenatal growth restriction (Ibáñez et al., 2006; Ong et al., 2000), which can result in part from the constraints of small maternal size due to the mother's own growth environment (Kramer, 1987; Ramakrishnan et al., 1999; Veena et al., 2004; Wells, 2010). Where environmental conditions change substantially over one or two generations, for example due to rural-urban migration or the nutritional transition, both linear growth and adiposity may be affected in young children, and this may account for direct associations between height and adiposity, as has been observed in various populations (Brophy et al., 2012; Franklin, 1999; Kain et al., 2005; Monteiro et al., 2003; Wells and Cole, 2011, in press).

These two different scenarios could explain why some studies demonstrate an association between short stature and adiposity among children (Fernald and Neufeld, 2006; Kruger et al., 2010; Popkin et al., 1996; Said-Mohamed et al., 2009; Steyn et al., 2005) and adults (Asao et al. 2006; Florencio et al., 2003; Leonard et al., 2009; Sichieri et al., 2010) while others show positive correlations between height and adiposity (Brophy et al., 2012; Franklin, 1999; Kain et al., 2005; Wells and Cole, in press), or no relationship (Cameron et al., 2005; Freedman et al., 2002; Mukuddem-Petersen and Kruger, 2004; Stanojevic et al., 2007; Walker et al., 2006). The dual burden is thus likely to be contingent on environment and population history (Stanojevic et al., 2007; Wells, 2012b; Wells and Cole, 2011).

However, methodological factors may also be relevant. Studies frequently analyze associations between height (or height *z* score) and adiposity indicators such as body mass index (BMI). When adiposity measures incorporate height in their denominator (e.g., BMI, waist circumference-height ratio (WCHtR)), a negative correlation between height and adiposity may be generated as an artifact of random measurement error in height when the true relationship is absent or even positive (Haaga, 1986; Timæus, 2012).

Although direct measures of adiposity (e.g., body composition measured by DXA, CT, MRI, or bioimpedance) are considered more accurate than proxies such as BMI or WCHtR, the required equipment is often unavailable in rural settings and LMICs where stunting is common, whereas weight, height, and waist circumference are more easily recorded. Using an additional measure of linear body size other than height in analyses using height-adjusted adiposity proxies like BMI would help to confirm that results are not biased by height measurement error. Tibia length (directly measured or using the proxy of knee height) potentially offers a good additional linear size indicator for assessing whether height measurement error may influence results based on BMI or WCHtR, as it is measured completely independently of height, unlike e.g. lower limb length, which is frequently calculated from sitting and standing heights. In addition, there is growing evidence that lower leg length, measured as tibia length or knee height, is a more sensitive indicator of poor growth than lower limb length or stature (Bailey et al., 2007; Bogin and Varela-Silva, [Bibr b6],[Bibr b7]; Lampl et al., 2003; Leitch, 1951; Pomeroy et al., 2012).

Understanding the circumstances under which the dual burden is observed in individual children will help elucidate its etiology and the conditions under which children are most at risk, and is critical for designing appropriate interventions to alleviate stunting without exposing already vulnerable populations to increased chronic disease in adulthood (Duran et al., 2006; Popkin et al., 1996; Varela-Silva et al., 2012; Victora, 2009). This study therefore has two objectives. First, we examine the individual-level dual burden among children from two low socioeconomic status (SES) populations in Peru, one from the urban lowlands and one from the rural highlands. As high and low altitude populations are characterized by complex ecological differences likely involving physical stresses, disease load, and diet and activity patterns (Masterson Creber et al., 2010; Niermeyer et al., 2009; Rivera-Ch et al., 2008), with some of these factors further incorporating intergenerational effects, we hypothesize that associations between height and adiposity might not be the same. Second, we aim to demonstrate how tibia length can be used to confirm that measurement error in height does not influence the results.

## SUBJECTS AND METHODS

A convenience sample of Peruvian children from two populations and aged between 6 months and 14 years participated in the study (*n* = 447). The first sample came from Pampas de San Juan de Miraflores, Lima (latitude −12.0, longitude −77.0; hereafter “lowlands”), a well-established but unplanned peri-urban settlement (shanty town) (Checkley et al., 2002; Miranda et al., [Bibr b54],[Bibr b55]) with an estimated population of 40,000 at the turn of the millennium (Checkley et al., 2002), but which has continued to grow since. The second sample came from various small, relatively isolated rural communities in the Santillana and Vinchos Districts of Ayacucho Region at 3,100–4,400 m altitude (latitude −13.2, longitude −74.2 for Ayacucho city; hereafter “highlands”: Supporting Information Fig. 1). In 2007, the populations of Santillana and Vinchos Districts were reportedly 7,000 and 16,000, respectively (INEI 2009; ODEI—Ayacucho 2008).

Both lowland and highland children are at risk of stunting due to low SES (Checkley et al., 1998; INEI, 2009; Sterling et al., 2012). However, their environments differ significantly in ways that may influence the risk of both obesity and stunting. High altitude is frequently characterized as a “multi-stress” environment, where people typically experience greater cold and aridity, lower oxygen availability, poorer diets, more limited access to healthcare and education, and high levels of physical activity (Niermeyer et al., 2009; Rivera-Ch et al., 2008). Previous studies have interpreted the slower growth and shorter stature of highland populations compared with their lowland counterparts as reflecting the impacts of these stressors (e.g., Beall et al., 1977; Dittmar, 1997; Frisancho, 1976; Greksa, 2006; Leonard et al., 1990; Pawson, 1976; Pawson and Huicho, 2010; Pomeroy et al., 2012). Thus, highland children may be predicted to be at greater risk of stunting than lowland children.

In terms of obesity risk, rural highland children are likely to fare better than their lowland counterparts in that urban environments in South America (as elsewhere in the world) are typically associated with higher fat and sugar consumption and reduced physical activity levels, factors linked to increased obesity risk (Dufour and Piperata, 2004; Fraser, 2005; Jacoby et al., 2003; Masterson Creber et al., 2010). Although we have no empirical data on diet and activity in our study sample, our observations in the field suggest marked differences between the populations consistent with urban-rural contrasts reported elsewhere in South America. Highland children often walked long distances to school, assisted with subsistence tasks including herding and gathering firewood, and consumed a more traditional diet than their lowland counterparts.

Consistent with our observations on activity among these children, a study of adults from the same lowland and highland communities demonstrated markedly lower physical activity levels in the urban lowlands along with greater levels of obesity (Masterson Creber et al., 2010). They reported that the World Health Organization (WHO) age-standardized prevalence of low physical activity was 2% among rural adults, compared with 32% and 39% in rural-urban migrants and rural residents, respectively.

In addition, greater cold exposure among highland children could influence fat distribution. Previous work has shown a tendency for greater overall adiposity (Beall and Goldstein, 1992; Wells, 2012a) and perhaps greater abdominal adiposity in populations from cold climates (Beall and Goldstein, 1992; though see Wells, 2012a). Variation in fat distribution has also been suggested to reflect differences in pathogen load between populations (Wells and Cortina-Borja, 2013). Although we are not aware of good data comparing infectious disease loads in similar populations to those studied here, respiratory infections are reportedly more frequent in the highlands (Way, 1976), suggesting differences in pathogen profiles as well as access to healthcare. Finally, intergenerational effects acting through epigenetic mechanisms or the influence of maternal phenotype on prenatal and early postnatal growth may also influence offspring height and body composition, particularly where low birth weight is associated with exposure to an obesogenic environment (Wells, 2010).

In the lowland study site, households with children of appropriate ages were identified from a door-to-door survey conducted as part of the PERU MIGRANTS study (Miranda et al., 2009) and were approached to participate. In the highlands, different strategies were pursued according to the size and location of the community, including door-to-door enquiry and identifying potential participants with the assistance of teachers and healthcare workers living in those communities. Written informed consent was obtained from a parent or legal guardian, and participants aged 6 years or over gave their assent. Date of birth was confirmed from official birth or identification documents, or school records. One child per household was included, and participation was voluntary. Participants were born and raised in the study region and were not affected by chronic medical conditions (aside from nutritional problems) that might affect growth. The study received ethical approval from the Institutional Ethics Committee at the Universidad Peruana Cayetano Heredia, Lima, and the Health Directorate for Ayacucho Region (Dirección Régional de Salud Ayacucho, DIRESA).

Anthropometry was measured by a single trained observer (EP) using standard methods (Cameron, 2004; Lohman et al., 1988). Height was measured to the nearest mm as recumbent length in individuals under two years of age using a Rollametre (Dunmow, UK), and as standing height in those aged over two years using a Leicester Height Measure (Seca). Tibia length was measured to the nearest mm using sliding callipers (Cameron, 2004). Weight was measured to the nearest 100 g using Tanita 352 scales (Tanita, Japan). Children were weighed in light clothes, and adjustments made based on the known weights of standard clothing items. Umbilical waist circumference was measured using a 15 mm-wide non-stretch fiberglass tape (Hoechtmass, Germany).

*Z* scores for height-, weight- and BMI-for-age and weight-for-height were calculated based on the WHO standards (WHO Multicentre Growth Reference Study Group, 2006) and references (de Onis et al., 2007) for children aged under and over 5 years, respectively. As there are no reference data for tibia length, sex-specific internal *z* scores were calculated for stature, tibia length, and BMI in the combined lowland and highland sample after fitting centiles using the LMS method (Cole, 1990; Cole and Green, 1992).

To characterize the study samples, the percentage of stunted children was calculated following the WHO definition (height-for-age *z* score < −2: WHO Expert Committee on Physical Status, 1995). For BMI-for-age, thresholds for overweight and obese followed the International Obesity Task Force (IOTF) recommendations (Cole et al., 2000; de Onis et al., 2007). The numbers of children who were simultaneously stunted and overweight or obese (i.e., showed individual “dual burden”) were calculated. The IOTF cut-offs are only available from age 2 onward, so the youngest age group was excluded from these analyses (*n* = 86). The proportion of children in each sample with a WCHtR above the recommended threshold (Browning et al., 2010) of 0.5 is also presented.

Frequencies of stunting and underweight/overweight included all the study participants to characterize the study sample more completely. As height growth appears to become largely canalized by 2–3 years of age (Dewey and Adu-Afarwuah, 2008; Martorell et al., 1994; Mei et al., 2004; Schroeder et al., 1995; Stein et al., 2010) and patterns of weight gain, adiposity, and body composition are complex and transient during puberty, the analyses of the relationship between height and adiposity were restricted to children aged 3–8.5 years (*n* = 201). Although pubertal onset was not assessed directly, the vast majority of children aged below 8.5 years were prepubertal in a similar low SES population in the Americas (Wilson et al., 2011).

To assess the relationship between linear body size (height or tibia length) and adiposity indicators, linear regression was performed of WHO BMI *z* score on WHO height *z* score, and of internal BMI *z* score on internal height or tibia length *z* score. WCHtR was regressed on WHO height, internal height, or internal tibia length *z* score. For height, both WHO and internal *z* scores were analyzed to confirm that the results did not differ according to how the *z* scores were derived. Study site and the interaction between study site and height or tibia length *z* score (as appropriate) were included in the models to test for differences in the relationship between adiposity and linear body size measures between the samples. For WCHtR, age and sex were included in the model since WCHtR was unadjusted for these factors.

To demonstrate the effects of different levels of height measurement error on the association between stature and BMI (Haaga, 1986; Timæus, 2012), random standard normal deviates were generated, multiplied by various levels of measurement error (2, 5, or 10 mm), and added to the original height measurement. BMI was recalculated with the new height measurement, and *z* scores for the new height and BMI were calculated using the WHO data. Analyses were conducted in SPSS v. 21.0, and *P* < 0.05 was considered significant.

## RESULTS

Supporting Information Table 1 gives sample sizes and summary statistics for the study samples by age group, sex, and population. Stunting was far more prevalent in the highlands than the lowlands (Supporting Information Fig. 2). Highland stunting rates exceeded 40% while in the lowlands, stunting rates were 2% among infants and children, rising to 8% for the oldest age group, which might reflect delayed maturation compared with the reference population. In both samples, the majority of children had a “normal” BMI by the IOTF criteria (Supporting Information Fig. 3). The frequency of overweight or obesity was highest in the lowlands (∼30%), while even in the highlands a few children (< 10%) were overweight between 2 and 8.5 years. Despite relatively high levels of stunting in the highlands, few children were classed as thin according to the IOTF criteria. Thinness was also uncommon in the lowlands. One of the 76 stunted highland children and two of the eight stunted lowland children were overweight, and none were obese (Table1). WCHtR was generally high, exceeding the 0.5 cut-off in more than half of both the highland and lowland samples. The only exception was for the oldest highland children where 25% had a WCHtR over 0.5 (Supporting Information Fig. 4).

**Table 1:** Frequency of stunted and non-stunted children in “normal,” “overweight,” and “obese” categories based on BMI, using the International Obesity Task Force definitions and WHO reference data

Sample	Stunted (height *z* score <−2)	IOTF BMI-for-age category (*n*)				
		Thin (Grade 1–2)	“Normal”	Overweight	Obese	Total
Lowland	No	4	134	36	19	193
	Yes	1	5	2	0	8
Highland	No	4	77	3	0	84
	Yes	7	68	1	0	76
Total		16	284	42	19	361

**Figure 1:**
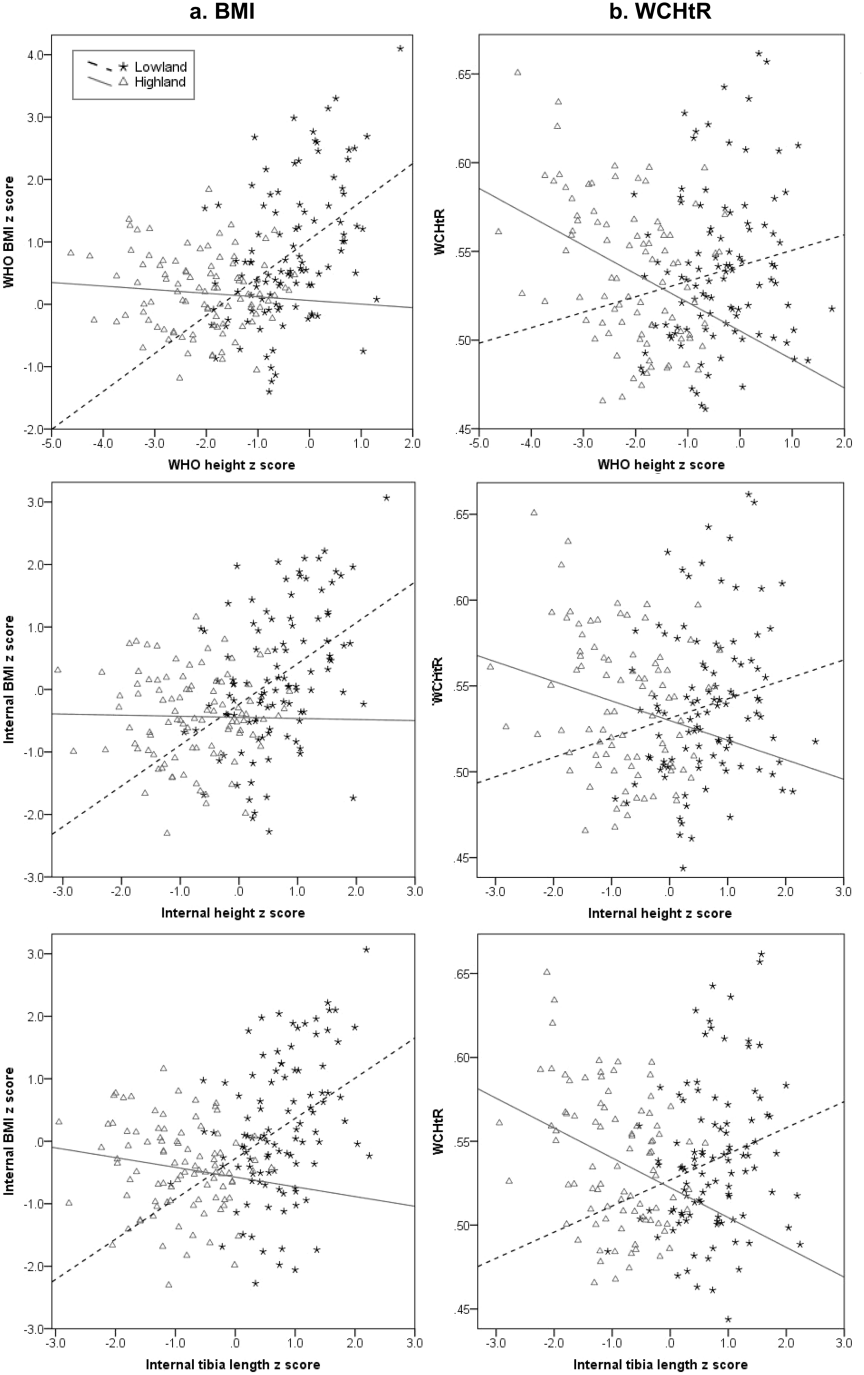
Scatterplots of BMI *z* scores (a) and waist circumference-height ratio (WCHtR: b) against height and tibia length *z* scores. Interaction for height or tibia length *z* score and site (highland or lowland) is highly significant (≤0.001 in all cases).

Highland and lowland children showed contrasting relationships between linear body size and proxies for adiposity, as indicated by significant interaction terms between site and height or tibia length in all models (*P* < 0.001). The two proxies for adiposity also showed different relationships with linear body size in the two samples (Tables2 and 3, Fig. 1). There was little relationship between BMI and height or tibia length among the highland children, while there was a clear positive association among the lowland children (Table2, Fig. 1a). In contrast, the relationship between WCHtR and height or tibia length was weakly positive among lowland children and strongly negative among highland children (Table3, Fig. 1b).

**Table 2:** Regression models for BMI z score on height or tibia length z scores for children aged 3–8.5 years

Analysis	Regression term	Unstandardized coefficients		*β*	*P*	Adjusted *R*^2^
		*B*	SE			
WHO BMI and height *z* scores	Constant	1.04	0.09	–	<0.001	0.231
	Site = highland	−0.98	0.23	−0.51	<0.001	
	WHO height *z*	0.61	0.10	0.78	<0.001	
	Site ^*^ WHO height *z*	−0.67	0.13	−0.87	<0.001	
Internal BMI and height *z* scores	(Constant)	−0.27	0.12	–	0.05	0.202
	Site = highland	−0.21	0.18	−0.10	0.2	
	Internal height *z*	0.65	0.13	0.70	<0.001	
	Site ^*^ Internal height *z*	−0.67	0.17	−0.46	<0.001	
Internal BMI and tibia length *z* scores	(Constant)	−0.28	0.14	–	0.05	0.183
	Site = highland	−0.29	0.20	−0.15	0.2	
	Internal tibia length *z*	0.64	0.14	0.71	<0.001	
	Site ^*^ Internal tibia length *z*	−0.80	0.19	−0.56	<0.001	

SE = standard error.

**Table 3:** Regression models for WCHtR on height or tibia length z scores for children aged 3–8.5 years

Analysis	Regression term	Unstandardized coefficients		*β*	*P*	*R*^2^
		*B*	SE			
WCHtR and WHO height *z* score	Constant	0.61	0.01	–	<0.001	0.280
	Age	−0.01	0.001	−0.46	<0.001	
	Sex = male	−0.01	0.005	−0.16	0.01	
	Site = highland	−0.04	0.01	−0.44	<0.001	
	WHO height *z*	0.01	0.005	0.31	0.02	
	Site ^*^ WHO height z	−0.03	0.006	−0.72	<0.001	
WCHtR and internal height *z* score	(Constant)	0.60	0.01	–	<0.001	0.258
	Age	−0.01	0.001	−0.47	<0.001	
	Sex = male	−0.01	0.005	−0.16	0.01	
	Site = highland	−0.01	0.008	−0.09	0.3	
	Internal height *z*	0.01	0.005	0.26	0.04	
	Site ^*^ Internal height *z*	−0.02	0.007	−0.38	0.001	
WCHtR and internal tibia length *z* score	(Constant)	0.60	0.01	–	<0.001	0.270
	Age	−0.01	0.001	−0.46	<0.001	
	Sex = male	−0.01	0.005	−0.14	0.03	
	Site = highland	−0.001	0.009	−0.10	0.3	
	Internal tibia length *z*	0.01	0.006	0.36	0.02	
	Site ^*^ Internal tibia length *z*	−0.03	0.008	−0.48	<0.001	

SE = standard error.

Results for the regression of internal BMI on internal tibia length were similar to those for BMI on height (Table2, Fig. 1a). In all models, the regression coefficients for both stature and tibia length were highly significant and similar in magnitude (*B* = 0.61–0.65, *R*^2^ = 0.18–0.23). Thus, the analysis of tibia length confirms the validity of the results using stature in this dataset. Similarly, analyses of WCHtR and tibia length did not differ greatly from analyses using stature (Table3, Fig. 1b), again supporting the validity of the results based on height.

**Figure 2:**
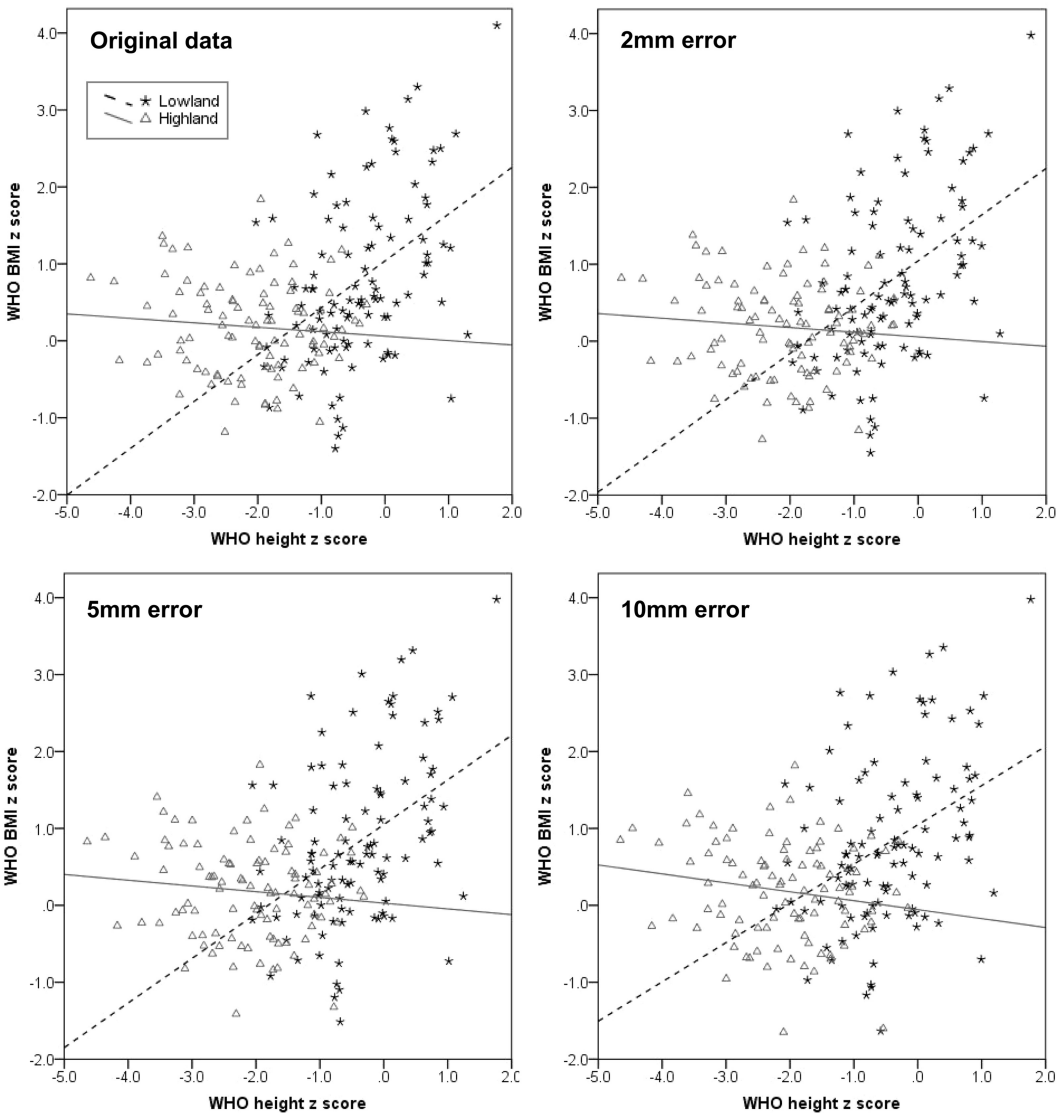
Scatterplots of BMI *z* score against height *z* score demonstrating the impact of increasing measurement error in height on the stature-BMI relationship.

Consistent with this finding, adding increasing measurement error to the height data resulted in a progressive decrease in the regression coefficient of BMI on height as predicted (Fig. 2, Table4), but even where a large random measurement error of 10 mm was added, the pattern of the relationship between WHO height and BMI *z*-scores remained unchanged. Thus, the pattern of association of BMI and height is very unlikely to be due to measurement error in height.

**Table 4:** Results of regression models for WHO BMI z score on WHO height z score for children aged 3–8.5 years, with the progressive addition of greater measurement error

Measurement error added to height (mm)	Regression term	Unstandardized Coefficients		*β*	*P*	Adjusted *R*^2^
		*B*	SE			
0	Constant	1.04	0.09	–	<0.001	0.231
	Site = highland	−0.98	0.23	−0.51	<0.001	
	WHO height *z*	0.61	0.10	0.78	<0.001	
	Site ^*^ WHO height *z*	−0.67	0.14	−0.87	<0.001	
2	(Constant)	1.05	0.09	–	<0.001	0.231
	Site = highland	−1.00	0.23	−0.52	<0.001	
	WHO height *z*	0.60	0.10	0.77	<0.001	
	Site ^*^ WHO height *z*	−0.66	0.14	−0.87	<0.001	
5	(Constant)	1.05	0.09	–	<0.001	0.225
	Site = highland	−1.02	0.23	−0.534	<0.001	
	WHO height *z*	0.58	0.10	0.75	<0.001	
	Site ^*^ WHO height *z*	−0.66	0.14	−0.85	<0.001	
10	(Constant)	1.05	0.10	–	<0.001	0.202
	Site = highland	−1.10	0.24	−0.567	<0.001	
	WHO height *z*	0.51	0.10	0.65	<0.001	
	Site ^*^ WHO height *z*	−0.63	0.14	−0.81	<0.001	

SE = standard error.

## DISCUSSION

Our data support proposals that there is no simple relationship between stunting and overweight or obesity risk among Peruvian children, as highland and lowland children showed contrasting patterns in the relationship between height and adiposity measures (BMI, WCHtR). Low SES urban lowland children showed low levels of stunting, but higher levels of obesity and an increase in BMI with increasing stature. In contrast, rural highland children showed higher levels of stunting with low levels of either thinness or overweight, and no association between BMI and linear growth measurements, but an inverse relationship between WCHtR and height.

Although further data are required to elucidate the relationship between environmental factors and the dual burden, we propose one explanation for our results. These two populations are exposed to different environments which are likely to present different opportunities for catch-up growth and for the accrual of excess adipose tissue. The lowland pattern would be consistent with the model whereby children that have undergone the most rapid postnatal growth are both taller and have greater adiposity (Franklin, 1999; Wells and Cole, 2011, in press). Alternatively, as growth was generally better and socioeconomic variation greater among lowland children than among highland children (see also Pomeroy et al., 2012), the positive relationship between BMI and stature for the lowland sample may exist because some children may have had consistently higher levels of nutrition resulting in taller height and greater weight through childhood.

The weaker association between WCHtR and height among the lowland children may indicate that much of the positive association between BMI and growth is due to lean mass index (lean mass relative to height) rather than adiposity. BMI *z*-score may be more sensitive to variability in lean mass than is WCHtR, hence, BMI may be reflecting lean mass index as well as adiposity (Wells, 2000). Therefore, WCHtR may be a more reliable indicator of abdominal adiposity, and it is more closely related to various indicators of metabolic disease risk among adults and children (Browning et al., 2010).

In contrast, the highland children may conform more closely to the pattern whereby stunting is associated with altered metabolism and fat distribution (Hoffman et al., [Bibr b36],[Bibr b35]; Leonard et al., 2009; Martins et al., 2004; Martins and Sawaya, 2006; Mukuddem-Petersen and Kruger, 2004; Wilson et al., 2012). The lack of relationship between BMI and stature among highland children may result from a marginal diet and high activity levels that preclude the accumulation of extra body mass across the range of height. However, the elevated waist circumference relative to stature among highland children who were shorter for their age is consistent with a tendency for central adiposity (Mukuddem-Petersen and Kruger, 2004; Walker et al., 2002). Direct measurements of body composition and fat distribution are needed to confirm that elevated WCHtR indicates a more centralized fat distribution among these children.

We used two different approaches to avoid the possibility that associations between height and adiposity might emerge as an artifact of height measurement error. Although such artifacts have been proposed previously (Haaga, 1986; Timæus, 2012), our results indicate that the magnitude of this effect is modest. We further demonstrated that our findings are similar whether we indexed growth status through height *z*-score, or tibia *z*-score, where measurement error is independent of the adiposity outcome and therefore unable to generate autocorrelations. Application of our approach involving both stature and tibia length (or knee height) measurements could help to clarify why the relationship between stature and adiposity varies between studies, and to exclude this methodological explanation.

Other methodological problems, such as differing definitions of stunting and obesity, could contribute to the varied results between studies (Flegal and Ogden, 2011; Freedman and Sherry, 2009). BMI thresholds for “overweight” and “obese” are not derived from associations with disease risk for children, unlike those for adults. They are either derived so that by 18 years of age the thresholds correspond to those of 25 and 30 kg/m^2^ defined for adults based on disease risk (IOTF: Cole et al., 2000), or defined arbitrarily (National Center for Health Statistics, NCHS: Must et al., 1991; Ogden and Flegal, 2010). Definitions of stunting are similarly arbitrary and differ between the WHO (WHO Expert Committee on Physical Status, 1995) and US Centers for Disease Control (Frisancho, 2008) guidelines, so the prevalence of the dual burden varies depending on the criteria and reference data used (Varela-Silva et al., 2012).

In addition, stunting according to the WHO criteria is defined purely statistically as height-for-age below −2 *z* scores, which approximates 2.5% of a normally distributed sample. So by definition 2.5% of the WHO reference sample, purportedly well-nourished and healthy, would still be classed as stunted, and we can expect similar “stunting” rates in other well-nourished, healthy samples. The majority of these “stunted” children may be physiologically normal and just fall at the lower extreme of normal height variation, meaning there may be no relationship between stunting and obesity risk. Such definitional problems may be avoided by analyzing the relationship between stature and adiposity across the full data range as we have done, which is preferable as the relationship between health risks and short stature apply linearly across the height range (Varela-Silva et al., 2012).

A limitation of our study is that we have no direct data on dietary intake, activity levels, infection rates, cold exposure, or parental phenotype that would help us to interpret the results and suggest with more confidence why we observed differences between highland and lowland children in the relationship between stunting and adiposity. Furthermore, our sample was relatively small, limiting our ability to examine the causes of variation in stature and adiposity within populations or between the sexes and at different ages. Nonetheless, our results offer interesting insight into the pattern of population differences in stature and adiposity during infancy and childhood, and demonstrate that contrasting relationships may occur in subpopulations living in differing environments.

In conclusion, our results suggest that urban lowland and rural highland Peruvian children of low SES differ in their patterns of stunting and adiposity as assessed by BMI and WCHtR. Lowland children were rarely stunted but more likely to be obese. There was a positive relationship between height and BMI, but the increase in WCHtR with height was less marked. This contrasts markedly with rural highland children, who were more likely to be stunted and showed little change in BMI but a decrease in WCHtR with increasing height. The different associations between height and adiposity in these two sub-populations support the notion that there is no simple association between growth rate and obesity, and that the association between these traits is context-specific (Wells, 2012b). Elucidating the complexity of the height–adiposity relationship is challenging, but this study also demonstrates how one methodological problem, that of measurement error in height affecting some proxies for adiposity, can be discounted by the using an additional independent linear body size measurement, such as tibia length or knee height.
