# Supplementary material for: Stunting, adiposity, and the individual-level “dual burden” among urban lowland and rural highland peruvian children
Source: Am J Hum Biol. 2014 Apr 7;26(4):481–90. doi: 10.1002/ajhb.22551 (PMC4312888; doi:10.1002/ajhb.22551)
Supplement: Supplementary file 1 [file ajhb0026-0481-sd1.doc]

Stunting, adiposity and the individual-level ‘dual burden’ among urban lowland and rural highland Peruvian children

**Authors:** Emma Pomeroy ^a,b^

Jay T. Stock ^b^

Sanja Stanojevic ^c^

J. Jaime Miranda ^d^

Tim J. Cole ^e^

Jonathan C.K. Wells ^f^

^a^ Newnham College, University of Cambridge, UK

^b^ Division of Biological Anthropology, Department of Archaeology and Anthropology, University of Cambridge, UK

^c^ Division of Respiratory Medicine, The Hospital for Sick Children, Toronto, Ontario, Canada

^d^ CRONICAS Centre of Excellence in Chronic Diseases and Department of Medicine, School of Medicine, Universidad Peruana Cayetano Heredia, Lima, Peru

^e^ Centre for Paediatric Epidemiology and Biostatistics, Institute of Child Health, University College London, UK

^f^ Childhood Nutrition Research Centre, Institute of Child Health, University College London, UK

Short title: Stunting and adiposity among Peruvian children

Corresponding author:

Dr Emma Pomeroy Tel: +44 7815 189784

Newnham College Email: emma.pomeroy@cantab.net

Cambridge, CB3 9DF, UK

**Funding:** This work was supported by the Arts and Humanities Research Council (UK) (PhD funding to EP); the University of Cambridge Centre for Latin American Studies Abbey-Santander Travel Grants (to EP); Newnham College, Cambridge (Junior Research Fellowship to EP); the National Institutes of Health (contract HHSN268200900033C to JJM); and the Medical Research Council (grant MR/J004839/1 to TJC).

**Abstract**

Background: The causes of the ‘dual burden’ of stunting and obesity remain unclear, and its existence at the individual level varies between populations. We investigate whether the individual dual burden differentially affects low socioeconomic status Peruvian children from contrasting environments (urban lowlands, rural highlands), and whether tibia length can discount the possible autocorrelation between adiposity proxies and height due to height measurement error

Methods: Stature, tibia length, weight, and waist circumference were measured in children aged 3-8.5 years (n=201). Height and body mass index (BMI) z scores were calculated using international reference data. Age-sex-specific centile curves were also calculated for height, BMI and tibia length. Adiposity proxies (BMI z score, waist circumference-height ratio (WCHtR)) were regressed on height and also on tibia length z score,

Results: Regression model interaction terms between site (highland vs. lowland) and height indicate that relationships between adiposity and linear growth measures differed significantly between samples (p<0.001). **H**eight was positively associated with BMI among urban lowland children, and more weakly with WCHtR. Among rural highland children, height was negatively associated with WCHtR but unrelated to BMI. Similar results using tibia length rather than stature indicate that stature measurement error was not a major concern.

Conclusions: Lowland and rural highland children differ in their patterns of stunting, BMI and WCHtR. These contrasts likely reflect environmental differences and overall environmental stress exposure. Tibia length or knee height can be used to assess the influence of measurement error in height on the relationship between stature and BMI or WCHtR.

Key words: dual burden; stunting; obesity; measurement error; tibia length

Introduction

Amongst the myriad factors that contribute to obesity risk, a number of studies have reported an association between stunting and excess adiposity (Varela-Silva et al. 2012). This so-called ‘dual burden’ of malnutrition is particularly relevant in low-middle income countries (LMICs: Black et al. 2013; Popkin et al. 1996; Varela-Silva et al. 2012; Victora et al. 2008), where low birth weight and poor growth often exist alongside a transition to more sedentary lifestyles and westernized diets. The mechanisms underlying this association between stunting and obesity and indeed the extent to which the dual burden exists at the individual level remain contentious, but are important for devising strategies to reduce the health and economic burdens of obesity.

Stunting and overweight might coexist within individuals because overweight can develop rapidly, whereas the resolution of height deficits from chronic malnutrition may take several generations (Wells and Stock 2011). Alternatively, there is some evidence that stunted children have an altered body composition and fat distribution (Hoffman et al. 2007; Martins et al. 2004; Mukuddem-Petersen and Kruger 2004; Wilson et al. 2012) that predisposes them to excess adiposity and abdominal fat distribution. This may be due to greater insulin sensitivity (Martins and Sawaya 2006) and/or reduced fat oxidation (Hoffman et al. 2000; Leonard et al. 2009) among stunted individuals (although see Said-Mohamed et al. 2012; Wren et al. 1997).

Rapid postnatal growth is also associated with greater adiposity (Chomtho et al. 2008; Dulloo et al. 2006; Howe et al. 2010; Ibáñez et al. 2006; Modi et al. 2006; Monasta et al. 2010; Ong and Loos 2006; Victora et al. 2007; Wells et al. 2007), so under some circumstances taller children, who have undergone the most rapid postnatal growth, may be at greater risk of obesity (Wells and Cole 2011). Rapid postnatal growth may be a ‘catch-up’ response to prenatal growth restriction (Ibáñez et al. 2006; Ong et al. 2000), which can result in part from the constraints of small maternal size due to the mother’s own growth environment (Kramer 1987; Ramakrishnan et al. 1999; Veena et al. 2004; Wells 2010). Where environmental conditions change substantially over one or two generations, for example due to rural-urban migration or the nutritional transition, both linear growth and adiposity may be affected in young children, and this may account for direct associations between height and adiposity, as has been observed in various populations (Brophy et al. 2012; Franklin 1999; Kain et al. 2005; Monteiro et al. 2003; Wells and Cole 2011; Wells and Cole In press).

These two different scenarios could explain why some studies demonstrate an association between short stature and adiposity among children (Fernald and Neufeld 2006; Kruger et al. 2010; Popkin et al. 1996; Said-Mohamed et al. 2009; Steyn et al. 2005) and adults (Asao et al. 2006; Florencio et al. 2003; Leonard et al. 2009; Sichieri et al. 2010) while others show positive correlations between height and adiposity (Brophy et al. 2012; Franklin 1999; Kain et al. 2005; Wells and Cole In press), or no relationship (Cameron et al. 2005; Freedman et al. 2002; Mukuddem-Petersen and Kruger 2004; Stanojevic et al. 2007; Walker et al. 2006). The dual burden is thus likely to be contingent on environment and population history (Stanojevic et al. 2007; Wells 2012b; Wells and Cole 2011).

However, methodological factors may also be relevant. Studies frequently analyze associations between height (or height z score) and adiposity indicators such as body mass index (BMI). When adiposity measures incorporate height in their denominator (e.g. BMI, waist circumference-height ratio (WCHtR)), a negative correlation between height and adiposity may be generated as an artifact of random measurement error in height when the true relationship is absent or even positive (Haaga 1986; Timæus 2012).

Although direct measures of adiposity (e.g. body composition measured by DXA, CT, MRI, or bioimpedance) are considered more accurate than proxies such as BMI or WCHtR, the required equipment is often unavailable in rural settings and LMICs where stunting is common, whereas weight, height and waist circumference are more easily recorded. Using an additional measure of linear body size other than height in analyses employing height-adjusted adiposity proxies like BMI would help to confirm that results are not biased by height measurement error. Tibia length (directly measured or using the proxy of knee height) potentially offers a good additional linear size indicator for assessing whether height measurement error may influence results based on BMI or WCHtR, since it is measured completely independent of height, unlike e.g. lower limb length, which is frequently calculated from sitting and standing heights. In addition there is growing evidence that lower leg length, measured as tibia length or knee height, is a more sensitive indicator of poor growth than lower limb length or stature (Bailey et al. 2007; Bogin and Varela-Silva 2008; Bogin and Varela-Silva 2010; Lampl et al. 2003; Leitch 1951; Pomeroy et al. 2012).

Understanding the circumstances under which the dual burden is observed in individual children will help elucidate its etiology and the conditions under which children are most at risk, and is critical for designing appropriate interventions to alleviate stunting without exposing already vulnerable populations to increased chronic disease in adulthood (Duran et al. 2006; Popkin et al. 1996; Varela-Silva et al. 2012; Victora 2009). This study therefore has two objectives. First we examine the individual-level dual burden among children from two low socioeconomic status (SES) populations in Peru, one from the urban lowlands and one from the rural highlands. As high and low altitude populations are characterized by complex ecological differences likely involving physical stresses, disease load, and diet and activity patterns (Masterson Creber et al. 2010; Niermeyer et al. 2009; Rivera-Ch et al. 2008), with some of these factors further incorporating intergenerational effects, we hypothesize that associations between height and adiposity might not be the same. Second, we aim to demonstrate how tibia length can be used to confirm that measurement error in height does not influence the results.

**Subjects and Methods**

A convenience sample of Peruvian children from two populations and aged between six months and 14 years participated in the study (n=447). The first sample came from Pampas de San Juan de Miraflores, Lima (latitude -12.0, longitude -77.0; hereafter ‘lowlands’), a well-established but unplanned peri-urban settlement (shanty town) (Checkley et al. 2002; Miranda et al. 2009; Miranda et al. 2011) with an estimated population of 40,000 at the turn of the millennium (Checkley et al. 2002), but which has continued to grow since. The second sample came from various small, relatively isolated rural communities in the Santillana and Vinchos Districts of Ayacucho Region at 3,100-4,400m altitude (latitude -13.2, longitude -74.2 for Ayacucho city; hereafter ‘highlands’: Supplementary Figure 1). In 2007, the populations of Santillana and Vinchos Districts were reportedly 7000 and 16,000 respectively (INEI 2009; ODEI - Ayacucho 2008).

Both lowland and highland children are at risk of stunting due to low SES (Checkley et al. 1998; INEI 2009; Sterling et al. 2012). However, their environments differ significantly in ways that may influence the risk of both obesity and stunting. High altitude is frequently characterized as a ‘multi-stress’ environment, where people typically experience greater cold and aridity, lower oxygen availability, poorer diets, more limited access to healthcare and education, and high levels of physical activity (Niermeyer et al. 2009; Rivera-Ch et al. 2008). Previous studies have interpreted the slower growth and shorter stature of highland populations compared with their lowland counterparts as reflecting the impacts of these stressors (e.g. Beall et al. 1977; Dittmar 1997; Frisancho 1976; Greksa 2006; Leonard et al. 1990; Pawson 1976; Pawson and Huicho 2010; Pomeroy et al. 2012). Thus highland children may be predicted to be at greater risk of stunting than lowland children.

In terms of obesity risk, rural highland children are likely to fare better than their lowland counterparts in that urban environments in South America (as elsewhere in the world) are typically associated with higher fat and sugar consumption and reduced physical activity levels, factors linked to increased obesity risk (Dufour and Piperata 2004; Fraser 2005; Jacoby et al. 2003; Masterson Creber et al. 2010). While we have no empirical data on diet and activity in our study sample, our observations in the field suggest marked differences between the populations consistent with urban-rural contrasts reported elsewhere in South America. Highland children often walked long distances to school, assisted with subsistence tasks including herding and gathering firewood, and consumed a more traditional diet than their lowland counterparts.

Consistent with our observations on activity among these children, a study of adults from the same lowland and highland communities demonstrated markedly lower physical activity levels in the urban lowlands along with greater levels of obesity (Masterson Creber et al. 2010). They reported that the World Health Organization (WHO) age-standardized prevalence of low physical activity was 2% among rural adults, compared with 32% and 39% in rural-urban migrants and rural residents respectively.

In addition, greater cold exposure among highland children could influence fat distribution. Previous work has shown a tendency for greater overall adiposity (Beall and Goldstein 1992; Wells 2012a) and perhaps greater abdominal adiposity in populations from cold climates (Beall and Goldstein 1992; though see Wells 2012a). Variation in fat distribution has also been suggested to reflect differences in pathogen load between populations (Wells and Cortina-Borja 2013). While we are not aware of good data comparing infectious disease loads in similar populations to those studied here, respiratory infections are reportedly more frequent in the highlands (Way 1976), suggesting differences in pathogen profiles as well as access to healthcare. Finally, intergenerational effects acting through epigenetic mechanisms or the influence of maternal phenotype on prenatal and early postnatal growth may also influence offspring height and body composition, particularly where low birth weight is associated with exposure to an obesogenic environment (Wells 2010).

In the lowland study site, households with children of appropriate ages were identified from a door-to-door survey conducted as part of the PERU MIGRANTS study (Miranda et al. 2009) and were approached to participate. In the highlands, different strategies were pursued according to the size and location of the community, including door-to-door enquiry and identifying potential participants with the assistance of teachers and healthcare workers living in those communities. Written informed consent was obtained from a parent or legal guardian, and participants aged 6 years or over gave their assent. Date of birth was confirmed from official birth or identification documents, or school records. One child per household was included, and participation was voluntary. Participants were born and raised in the study region and were not affected by chronic medical conditions (aside from nutritional problems) that might affect growth. The study received ethical approval from the Institutional Ethics Committee at the Universidad Peruana Cayetano Heredia, Lima, and the Health Directorate for Ayacucho Region (Dirección Régional de Salud Ayacucho, DIRESA).

Anthropometry was measured by a single trained observer (EP) using standard methods (Cameron 2004; Lohman et al. 1988). Height was measured to the nearest mm as recumbent length in individuals under two years of age using a Rollametre (Dunmow, UK), and as standing height in those aged over two years using a Leicester Height Measure (Seca). Tibia length was measured to the nearest mm using sliding callipers (Cameron 2004). Weight was measured to the nearest 100g using Tanita 352 scales (Tanita, Japan). Children were weighed in light clothes, and adjustments made based on the known weights of standard clothing items. Umbilical waist circumference was measured using a 15mm-wide non-stretch fiberglass tape (Hoechtmass, Germany).

Z scores for height-, weight- and BMI-for-age and weight-for-height were calculated based on the WHO standards (WHO Multicentre Growth Reference Study Group 2006) and references (de Onis et al. 2007) for children aged under and over 5 years respectively. As there are no reference data for tibia length, sex-specific internal z scores were calculated for stature, tibia length and BMI in the combined lowland and highland sample after fitting centiles using the LMS method (Cole 1990; Cole and Green 1992).

To characterize the study samples, the percentage of stunted children was calculated following the WHO definition (height-for-age z score <-2: WHO Expert Committee on Physical Status 1995). For BMI-for-age, thresholds for overweight and obese followed the International Obesity Task Force (IOTF) recommendations (Cole et al. 2000; de Onis et al. 2007). The numbers of children who were simultaneously stunted and overweight or obese (i.e. showed individual ‘dual burden’) were calculated. The IOTF cut-offs are only available from age 2 onwards, so the youngest age group was excluded from these analyses (n=86). The proportion of children in each sample with a WCHtR above the recommended threshold (Browning et al. 2010) of 0.5 is also presented.

Frequencies of stunting and underweight/overweight include all the study participants to characterize more completely the study sample. As height growth appears to become largely canalized by 2-3 years of age (Dewey and Adu-Afarwuah 2008; Martorell et al. 1994; Mei et al. 2004; Schroeder et al. 1995; Stein et al. 2010) and patterns of weight gain, adiposity and body composition are complex and transient during puberty, the analyses of the relationship between height and adiposity were restricted to children aged 3-8.5 years (n=201). Although pubertal onset was not assessed directly, the vast majority of children aged below 8.5 years were prepubertal in a similar low SES population in the Americas (Wilson et al. 2011).

To assess the relationship between linear body size (height or tibia length) and adiposity indicators, linear regression was performed of WHO BMI z score on WHO height z score, and of internal BMI z score on internal height or tibia length z score. WCHtR was regressed on WHO height, internal height or internal tibia length z score. For height, both WHO and internal z scores were analyzed to confirm that the results did not differ according to how the z scores were derived. Study site and the interaction between study site and height or tibia length z score (as appropriate) were included in the models to test for differences in the relationship between adiposity and linear body size measures between the samples. For WCHtR, age and sex were included in the model since WCHtR was unadjusted for these factors.

To demonstrate the effects of different levels of height measurement error on the association between stature and BMI (Haaga 1986; Timæus 2012), random standard normal deviates were generated, multiplied by various levels of measurement error (2, 5 or 10mm), and added to the original height measurement. BMI was recalculated with the new height measurement, and z scores for the new height and BMI were calculated using the WHO data. Analyses were conducted in SPSS v. 21.0, and p <0.05 was considered significant.

Results

Supplementary Table 1 gives sample sizes and summary statistics for the study samples by age group, sex and population. Stunting was far more prevalent in the highlands than the lowlands (Supplementary Figure 2). Highland stunting rates exceeded 40% while in the lowlands, stunting rates were 2% among infants and children, rising to 8% for the oldest age group, which might reflect delayed maturation compared with the reference population. In both samples the majority of children had a ‘normal’ BMI by the IOTF criteria (Supplementary Figure 3). The frequency of overweight or obesity was highest in the lowlands (~30%), while even in the highlands a few children (<10%) were overweight between 2 and 8.5 years. Despite relatively high levels of stunting in the highlands, few children were classed as thin according to the IOTF criteria. Thinness was also uncommon in the lowlands. One of the 76 stunted highland children and 2 of the 8 stunted lowland children were overweight, and none were obese (Table 1). WCHtR was generally high, exceeding the 0.5 cut-off in more than half of both the highland and lowland samples. The only exception was for the oldest highland children where 25% had a WCHtR over 0.5 (Supplementary Figure 4).

*** INSERT TABLE 1 HERE ***

Highland and lowland children showed contrasting relationships between linear body size and proxies for adiposity, as indicated by significant interaction terms between site and height or tibia length in all models (p<0.001). The two proxies for adiposity also showed different relationships with linear body size in the two samples (Tables 2 and 3, Figure 1). There was little relationship between BMI and height or tibia length among the highland children, while there was a clear positive association among the lowland children (Table 2, Figure 1a). In contrast, the relationship between WCHtR and height or tibia length was weakly positive among lowland children and strongly negative among highland children (Table 3, Figure 1b).

*** INSERT TABLES 2-3 AND FIGURE 1 HERE ***

Results for the regression of internal BMI on internal tibia length were similar to those for BMI on height (Table 2, Figure 1a). In all models, the regression coefficients for both stature and tibia length were highly significant and similar in magnitude (B = 0.61 to 0.65, R^2^ = 0.18 to 0.23). Thus the analysis of tibia length confirms the validity of the results using stature in this dataset. Similarly, analyses of WCHtR and tibia length did not differ greatly from analyses using stature (Table 3, Figure 1b), again supporting the validity of the results based on height.

Consistent with this finding, adding increasing measurement error to the height data resulted in a progressive decrease in the regression coefficient of BMI on height as predicted (Figure 2, Table 4), but even where a large random measurement error of 10mm was added, the pattern of the relationship between WHO height and BMI z-scores remained unchanged. Thus, the pattern of association of BMI and height is very unlikely to be due to measurement error in height.

*** INSERT FIGURE 2 & TABLE 4 HERE ***

**Discussion**

Our data support proposals that there is no simple relationship between stunting and overweight or obesity risk among Peruvian children, as highland and lowland children showed contrasting patterns in the relationship between height and adiposity measures (BMI, WCHtR). Low SES urban lowland children showed low levels of stunting, but higher levels of obesity and an increase in BMI with increasing stature. In contrast, rural highland children showed higher levels of stunting with low levels of either thinness or overweight, and no association between BMI and linear growth measurements, but an inverse relationship between WCHtR and height.

While further data are required to elucidate the relationship between environmental factors and the dual burden, we propose one explanation for our results. These two populations are exposed to different environments which are likely to present different opportunities for catch-up growth and for the accrual of excess adipose tissue. The lowland pattern would be consistent with the model whereby children that have undergone the most rapid postnatal growth are both taller and have greater adiposity (Franklin 1999; Wells and Cole 2011; Wells and Cole In press). Alternatively, as growth was generally better and socioeconomic variation greater among lowland children than among highland children (see also Pomeroy et al. 2012), the positive relationship between BMI and stature for the lowland sample may exist because some children may have had consistently higher levels of nutrition resulting in taller height and greater weight through childhood.

The weaker association between WCHtR and height among the lowland children may indicate that much of the positive association between BMI and growth is due to lean mass index (lean mass relative to height) rather than adiposity. BMI z-score may be more sensitive to variability in lean mass than is WCHtR, hence BMI may be reflecting lean mass index as well as adiposity (Wells 2000). Therefore WCHtR may be a more reliable indicator of abdominal adiposity, and it is more closely related to various indicators of metabolic disease risk among adults and children (Browning et al. 2010).

In contrast, the highland children may conform more closely to the pattern whereby stunting is associated with altered metabolism and fat distribution (Hoffman et al. 2007; Hoffman et al. 2000; Leonard et al. 2009; Martins et al. 2004; Martins and Sawaya 2006; Mukuddem-Petersen and Kruger 2004; Wilson et al. 2012). The lack of relationship between BMI and stature among highland children may result from a marginal diet and high activity levels that preclude the accumulation of extra body mass across the range of height. However, the elevated waist circumference relative to stature among highland children who were shorter for their age is consistent with a tendency for central adiposity (Mukuddem-Petersen and Kruger 2004; Walker et al. 2002). Direct measurements of body composition and fat distribution are needed to confirm that elevated WCHtR indicates a more centralized fat distribution among these children.

We used two different approaches to avoid the possibility that associations between height and adiposity might emerge as an artifact of height measurement error. Although such artifacts have been proposed previously (Haaga 1986; Timæus 2012), our results indicate that the magnitude of this effect is modest. We further demonstrated that our findings are similar whether we indexed growth status through height z-score, or tibia z-score, where measurement error is independent of the adiposity outcome and therefore unable to generate autocorrelations. Application of our approach involving both stature and tibia length (or knee height) measurements could help to clarify why the relationship between stature and adiposity varies between studies, and to exclude this methodological explanation.

Other methodological problems, such as differing definitions of stunting and obesity, could contribute to the varied results between studies (Flegal and Ogden 2011; Freedman and Sherry 2009). BMI thresholds for ‘overweight’ and ‘obese’ are not derived from associations with disease risk for children, unlike those for adults. They are either derived so that by 18 years of age the thresholds correspond to those of 25 and 30 kg/m^2^ defined for adults based on disease risk (IOTF: Cole et al. 2000), or defined arbitrarily (National Center for Health Statistics, NCHS: Must et al. 1991; Ogden and Flegal 2010). Definitions of stunting are similarly arbitrary and differ between the WHO (WHO Expert Committee on Physical Status 1995) and US Centers for Disease Control (Frisancho 2008) guidelines, so the prevalence of the dual burden varies depending on the criteria and reference data employed (Varela-Silva et al. 2012).

In addition, stunting according to the WHO criteria is defined purely statistically as height-for-age below -2 z scores, which approximates 2.5% of a normally distributed sample. So by definition 2.5% of the WHO reference sample, purportedly well-nourished and healthy, would still be classed as stunted, and we can expect similar ‘stunting’ rates in other well-nourished, healthy samples. The majority of these ‘stunted’ children may be physiologically normal and just fall at the lower extreme of normal height variation, meaning there may be no relationship between stunting and obesity risk. Such definitional problems may be avoided by analyzing the relationship between stature and adiposity across the full data range as we have done, which is preferable as the relationship between health risks and short stature apply linearly across the height range (Varela-Silva et al. 2012).

A limitation of our study is that we have no direct data on dietary intake, activity levels, infection rates, cold exposure or parental phenotype that would help us to interpret the results and suggest with more confidence why we observed differences between highland and lowland children in the relationship between stunting and adiposity. Furthermore, our sample was relatively small, limiting our ability to examine the causes of variation in stature and adiposity within populations or between the sexes and at different ages. Nonetheless, our results offer interesting insight into the pattern of population differences in stature and adiposity during infancy and childhood, and demonstrate that contrasting relationships may occur in subpopulations living in differing environments.

In conclusion, our results suggest that urban lowland and rural highland Peruvian children of low SES differ in their patterns of stunting and adiposity as assessed by BMI and WCHtR. Lowland children were rarely stunted but more likely to be obese. There was a positive relationship between height and BMI, but the increase in WCHtR with height was less marked. This contrasts markedly with rural highland children, who were more likely to be stunted and showed little change in BMI but a decrease in WCHtR with increasing height. The different associations between height and adiposity in these two sub-populations support the notion that there is no simple association between growth rate and obesity, and that the association between these traits is context-specific (Wells 2012b). Elucidating the complexity of the height-adiposity relationship is challenging, but the present study also demonstrates how one methodological problem, that of measurement error in height affecting some proxies for adiposity, can be discounted by the using an additional independent linear body size measurement, such as tibia length or knee height.

**Acknowledgements**

We are grateful to all the participants and their families who generously gave their time to take part in the study. We thank Lilia Cabrera and Angela Huamán Gómez of PRISMA, and all the field staff for their assistance in participant recruitment and data collection. Thanks to Dr. Antonio Bernabe Ortiz for his assistance in establishing the project, and to the Ayacucho Department Health Directorate (DIRESA) for their support. We thank the editor and two anonymous reviewers for their suggestions which helped significantly to improve the manuscript.

**Conflict of Interest**

The authors have no conflicts of interest to disclose.

**References**

Asao K, Kao WH, Baptiste-Roberts K, Bandeen-Roche K, Erlinger TP, Brancati FL. 2006. Short stature and the risk of adiposity, insulin resistance, and type 2 diabetes in middle age: the Third National Health and Nutrition Examination Survey (NHANES III), 1988-1994. Diabetes Care 29:1632-7.

Bailey SM, Xu J, Feng JH, Hu X, Zhang C, Qui S. 2007. Tradeoffs between oxygen and energy in tibial growth at high altitude. Am J Hum Biol 19:662-8.

Beall CM, Baker PT, Baker TS, Haas JD. 1977. The effects of high altitude on adolescent growth in southern Peruvian Amerindians. Hum Biol 49:109-24.

Beall CM, Goldstein MC. 1992. High prevalence of excess fat and central fat patterning among Mongolian pastoral nomads. Am J Hum Biol 4:747-756.

Black RE, Victora CG, Walker SP, Bhutta ZA, Christian P, de Onis M, Ezzati M, Grantham-McGregor S, Katz J, Martorell R and others. 2013. Maternal and child undernutrition and overweight in low-income and middle-income countries. Lancet 382:427-451.

Bogin B, Varela-Silva MI. 2008. Fatness biases the use of estimated leg length as an epidemiological marker for adults in the NHANES III sample. Int J Epidemiol 37:201-209.

Bogin B, Varela-Silva MI. 2010. Leg length, body proportion, and health: a review with a note on beauty. Int J Environ Res Public Health 7:1047-1075.

Brophy S, Rees A, Knox G, Baker J, Thomas NE. 2012. Child fitness and father’s BMI are important factors in childhood obesity: A school based cross-sectional study. PLoS One 7(5):e36597.

Browning LM, Hsieh SD, Ashwell M. 2010. A systematic review of waist-to-height ratio as a screening tool for the prediction of cardiovascular disease and diabetes: 0.5 could be a suitable global boundary value. Nutr Res Rev 23:247-269.

Cameron N. 2004. Measuring growth. In: Hauspie RC, Cameron N, Molinari L, editors. Methods in Human Growth Research. Cambridge: Cambridge University Press. p 68-107.

Cameron N, Wright MM, Griffiths PL, Norris SA, Pettifor JM. 2005. Stunting at 2 years in relation to body composition at 9 years in African urban children. Obesity 13:131-136.

Checkley W, Epstein LD, Gilman RH, Black RE, Cabrera L, Sterling CR. 1998. Effects of *Cryptosporidium parvum* infection in Peruvian children: Growth faltering and subsequent catch-up growth. Am J Epidemiol 148:497-506.

Checkley W, Gilman RH, Black RE, Lescano AG, Cabrera L, Taylor DN, Moulton LH. 2002. Effects of nutritional status on diarrhea in Peruvian children. J Pediatr 140:210-218.

Chomtho S, Wells JC, Williams JE, Davies PS, Lucas A, Fewtrell MS. 2008. Infant growth and later body composition: evidence from the 4-component model. The Am J Clin Nutr 87(6):1776-1784.

Cole, T.J. 1990. The LMS method for constructing normalized growth standards. Eur J Clin Nutr 44: 45-60.

Cole, TJ, Green, PJ. 1992. Smoothing reference centile curves: The lms method and penalized likelihood. Stat Med 11: 1305-1319.

Cole TJ, Bellizzi MC, Flegal KM, Dietz WH. 2000. Establishing a standard definition for child overweight and obesity worldwide: international survey. BMJ 320:1240.

de Onis M, Onyango AW, Borghi E, Siyam A, Nishidaa C, Siekmanna J. 2007. Development of a WHO growth reference for school-aged children and adolescents. Bull World Health Organ 85:660–667.

Dewey KG, Adu-Afarwuah S. 2008. Systematic review of the efficacy and effectiveness of complementary feeding interventions in developing countries. Mat Child Nutr 4 (Suppl. 1):24-85.

Dittmar M. 1997. Linear growth in weight, stature, sitting height and leg length, and body proportions of Aymara school-children living in an hypoxic environment at high altitude in Chile. Z Morphol Anthropol 81:333-44.

Dufour DL, Piperata BA. 2004. Rural-to-urban migration in Latin America: An update and thoughts on the model. Am J Hum Biol 16:395-404.

Dulloo AG, Jacquet J, Seydoux J, Montani JP. 2006. The thrifty `catch-up fat' phenotype: its impact on insulin sensitivity during growth trajectories to obesity and metabolic syndrome. Int J Obes 30(Suppl. 4):23-35.

Duran P, Caballero B, de Onis M. 2006. The association between stunting and overweight in Latin American and Caribbean preschool children. Food Nutr Bull 27:300-305.

Fernald LC, Neufeld LM. 2006. Overweight with concurrent stunting in very young children from rural Mexico: prevalence and associated factors. Eur J Clin Nutr 61:623-632.

Flegal KM, Ogden CL. 2011. Childhood obesity: are we all speaking the same language? Adv Nutr 2:159S-166S.

Florencio TT, Ferreira HS, Cavalcante JC, Luciano SM, Sawaya AL. 2003. Food consumed does not account for the higher prevalence of obesity among stunted adults in a very-low-income population in the Northeast of Brazil (Maceio, Alagoas). Eur J Clin Nutr 57:1437-1446.

Franklin M. 1999. Comparison of weight and height relations in boys from 4 countries. The Am J Clin Nutr 70:157S-162S.

Fraser B. 2005. Latin America's urbanisation is boosting obesity. Lancet 365:1995-1996.

Freedman DS, Khan LK, Mei Z, Dietz WH, Srinivasan SR, Berenson GS. 2002. Relation of childhood height to obesity among adults: the Bogalusa Heart Study. Pediatrics 109:e23.

Freedman DS, Sherry B. 2009. The validity of BMI as an indicator of body fatness and risk among children. Pediatrics 124 (Suppl. 1):S23-S34.

Frisancho AR. 1976. Growth and morphology at high altitude. In: Baker PT, Little MA, editors. Man in the Andes: A Multidisciplinary Study of High Altitude Quechua. Stroudsburg, Pennsylvania: Dowden, Hutchinson and Ross, Inc. p 180-207.

Frisancho AR. 2008. Anthropometric Standards: An Interactive Nutritional Reference of Body Size and Body Composition for Children and Adults. Ann Arbor: University of Michigan Press.

Greksa LP. 2006. Growth and development of Andean high altitude residents. High Alt Med Biol 7:116-24.

Haaga JG. 1986. Negative bias in estimates of the correlation between children's weight-for-height and height-for-age. Growth 50:147-154.

Hoffman DJ, Martins PA, Roberts SB, Sawaya AL. 2007. Body fat distribution in stunted compared with normal-height children from the shantytowns of São Paulo, Brazil. Nutrition 23:640-646.

Hoffman DJ, Sawaya AL, Verreschi I, Tucker KL, Roberts SB. 2000. Why are nutritionally stunted children at increased risk of obesity? Studies of metabolic rate and fat oxidation in shantytown children from São Paulo, Brazil. Am J Clin Nutr 72:702-707.

Howe LD, Tilling K, Benfield L, Logue J, Sattar N, Ness AR, Smith GD, Lawlor DA. 2010. Changes in ponderal index and body mass index across childhood and their associations with fat mass and cardiovascular risk factors at age 15. PLoS One 5:e15186.

Ibáñez L, Ong K, Dunger DB, de Zegher F. 2006. Early development of adiposity and insulin resistance after catch-up weight gain in small-for-gestational-age children. J Clin Endocrinol Metab 91:2153-2158.

INEI. 2009. Perfil Sociodemográfico del Departamento de Ayacucho. Ayacucho: Instituto Nacional de Estadística e Informática. Available online at <http://www.inei.gob.pe/Biblioinei4.asp>, accessed 19/05/2011.

Jacoby E, Bull F, Neiman A. 2003. Rapid changes in lifestyle make increased physical activity a priority for the Americas. Rev Panam Salud Pública 14:226-228.

Kain J, Uauy R, Lera L, Taibo M, Albala C. 2005. Trends in height and BMI of 6-year-old children during the nutrition transition in Chile. Obesity Res 13:2178-2186.

Kramer MS. 1987. Determinants of low birth weight: methodological assessment and meta-analysis. Bull World Health Organ 65):663-737.

Kruger HS, Pretorius R, Schutte AE. 2010. Stunting, adiposity, and low-grade inflammation in African adolescents from a township high school. Nutrition 26:90-99.

Lampl M, Kuzawa CW, Jeanty P. 2003. Prenatal smoke exposure alters growth in limb proportions and head shape in the midgestation human fetus. Am J Hum Biol 15:533-46.

Leitch I. 1951. Growth and health. Br J Nutr 5:142-51.

Leonard WR, Leatherman TL, Carey JW, Thomas RB. 1990. Contributions of nutrition vs. hypoxia to growth in rural Andean populations. Am J Hum Biol 2:613-626.

Leonard WR, Sorensen MV, Mosher MJ, Spitsyn V, Comuzzie AG. 2009. Reduced fat oxidation and obesity risks among the Buryat of Southern Siberia. Am J Hum Biol 21:664-670.

Lohman TG, Roche AF, Martorell R, editors. 1988. Anthropometric Standardization Reference Manual. Champaign, Illinois: Human Kinetics Books.

Martins PA, Hoffman DJ, Fernandes MTB, Nascimento CR, Roberts SB, Sesso R, Sawaya AL. 2004. Stunted children gain less lean body mass and more fat mass than their non-stunted counterparts: a prospective study. Br J Nutr 92:819-825.

Martins PA, Sawaya AL. 2006. Evidence for impaired insulin production and higher sensitivity in stunted children living in slums. Br J Nutr 95:996-1001.

Martorell R, Khan LK, Schroeder DG. 1994. Reversibility of stunting: epidemiological findings in children from developing countries. Eur J Clin Nutr 48 (Suppl. 1):S45-57.

Masterson Creber RM, Smeeth L, Gilman RH, Miranda JJ. 2010. Physical activity and cardiovascular risk factors among rural and urban groups and rural-to-urban migrants in Peru: a cross-sectional study. Rev Panam Salud Publica 28:1-8.

Mei Z, Grummer-Strawn LM, Thompson D, Dietz WH. 2004. Shifts in percentiles of growth during early childhood: Analysis of longitudinal data from the California Child Health and Development Study. Pediatrics 113:e617-627.

Miranda JJ, Gilman R, Garcia H, Smeeth L. 2009. The effect on cardiovascular risk factors of migration from rural to urban areas in Peru: PERU MIGRANT Study. BMC Cardiovasc Disord 9:23.

Miranda JJ, Gilman RH, Smeeth L. 2011. Differences in cardiovascular risk factors in rural, urban and rural-to-urban migrants in Peru. Heart 97:787-796.

Modi N, Thomas EL, Harrington TAM, Uthaya S, Dore CJ, Bell JD. 2006. Determinants of adiposity during preweaning postnatal growth in appropriately grown and growth-restricted term infants. Pediatr Res 60:345-348.

Monasta L, Batty GD, Cattaneo A, Lutje V, Ronfani L, Van Lenthe FJ, Brug J. 2010. Early-life determinants of overweight and obesity: a review of systematic reviews. Obes Rev 11:695-708.

Monteiro POA, Victora CG, Barros FC, Monteiro LMA. 2003. Birth size, early childhood growth, and adolescent obesity in a Brazilian birth cohort. Int J Obes 27: 1274-1282.

Mukuddem-Petersen J, Kruger HS. 2004. Association between stunting and overweight among 10-15-y-old children in the North West Province of South Africa: the THUSA BANA Study. Int J Obes Relat Metab Disord 28:842-851.

Must A, Dallal G, Dietz W. 1991. Reference data for obesity: 85th and 95th percentiles of body mass index (wt/ht2) and triceps skinfold thickness. Am J Clin Nutr 53:839-846.

Niermeyer S, Andrade Mollinedo P, Huicho L. 2009. Child health and living at high altitude. Arch Dis Child 94:806-811.

ODEI - Ayacucho. 2008. Ayacucho: Compendio Estadístico 2007-2008. Ayacucho: Oficina Departmental de Estadística e Informática de Ayacucho. Available online at http://www.inei.gob.pe/Biblioinei4.asp, accessed 19/05/2011.

Ogden CL, Flegal KM. 2010. Changes in terminology for childhood overweight and obesity. Hyattsville, MD: National Center for Health Statistics.

Ong KK, Loos RJF. 2006. Rapid infancy weight gain and subsequent obesity: Systematic reviews and hopeful suggestions. Acta Pædiatr 95:904-908.

Ong KKL, Ahmed ML, Emmett PM, Preece MA, Dunger DB. 2000. Association between postnatal catch-up growth and obesity in childhood: prospective cohort study. BMJ 320:967-971.

Pawson IG. 1976. Growth and development in high altitude populations: a review of Ethiopian, Peruvian, and Nepalese studies. Proc R Soc Lond B Biol Sci 194:83-98.

Pawson IG, Huicho L. 2010. Persistence of growth stunting in a Peruvian high altitude community, 1964-1999. Am J Hum Biol 22:367-374.

Pomeroy E, Stock JT, Stanojevic S, Miranda JJ, Cole TJ, Wells JCK. 2012. Trade-offs in relative limb length among Peruvian children: Extending the thrifty phenotype hypothesis to limb proportions. PLoS One 7:e51795.

Popkin BM, Richards MK, Montiero CA. 1996. Stunting is associated with overweight in children of four nations that are undergoing the nutrition transition. J Nutr 126:3009-3016.

Ramakrishnan U, Martorell R, Schroeder DG, Flores R. 1999. Role of intergenerational effects on linear growth. J Nutr 129:544.

Rivera-Ch M, Castillo A, Huicho L. 2008. Hypoxia and other environmental factors at high altitude. Int J Environ Health 2:92-106.

Said-Mohamed R, Allirot X, Sobgui M, Pasquet P. 2009. Determinants of overweight associated with stunting in preschool children of Yaoundé, Cameroon. Ann Hum Biol 36:146-161.

Said-Mohamed R, Bernard JY, Ndzana A-C, Pasquet P. 2012. Is overweight in stunted preschool children in Cameroon related to reductions in fat oxidation, resting energy expenditure and physical activity? PLoS ONE 7:e39007.

Schroeder DG, Martorell R, Rivera JA, Ruel MT, Habicht J-P. 1995. Age differences in the impact of nutritional supplementation on growth. J. Nutr. 125(4 (Suppl.)):1051-1059.

Sichieri R, dos Santos Barbosa F, Moura EC. 2010. Relationship between short stature and obesity in Brazil: a multilevel analysis. Br J Nutr 103:1534-1538.

Stanojevic S, Kain J, Uauy R. 2007. The association between changes in height and obesity in Chilean preschool children: 1996-2004. Obesity 15:1012-1022.

Stein AD, Wang M, Martorell R, Norris SA, Adair LS, Bas I, Sachdev HS, Bhargava SK, Fall CHD, Gigante DP and others. 2010. Growth patterns in early childhood and final attained stature: Data from five birth cohorts from low- and middle-income countries. Am J Hum Biol 22:353-359.

Sterling R, Miranda JJ, Gilman RH, Cabrera L, Sterling CR, Bern C, Checkley W. 2012. Early anthropometric indices predict short stature and overweight status in a cohort of peruvians in early adolescence. Am J Phys Anthropol 148:451-461.

Steyn NP, Labadarios D, Maunder E, Nel J, Lombard C. 2005. Secondary anthropometric data analysis of the National Food Consumption Survey in South Africa: The double burden. Nutrition 21:4-13.

Timæus IM. 2012. Stunting and obesity in childhood: a reassessment using longitudinal data from South Africa. Int J Epidemiol 41:764-772.

Varela-Silva MI, Dickinson F, Wilson H, Azcorra H, Griffiths PL, Bogin B. 2012. The nutritional dual-burden in developing countries--how is it assessed and what are the health implications? Coll Antropol 36:39-45.

Veena SR, Kumaran K, Swarnagowri MN, Jayakumar MN, Leary SD, Stein CE, Cox VA, Fall CHD. 2004. Intergenerational effects on size at birth in South India. Paediatr Perinat Epidemiol 18:361-370.

Victora CG. 2009. Nutrition in early life: a global priority. Lancet 374:1123-1125.

Victora CG, Adair L, Fall C, Hallal PC, Martorell R, Richter L, Sachdev HS. 2008. Maternal and child undernutrition: consequences for adult health and human capital. Lancet 371:340-357.

Victora CG, Sibbritt D, Horta BL, Lima RC, ScD TC, Wells J. 2007. Weight gain in childhood and body composition at 18 years of age in Brazilian males. Acta Pædiatr 96:296-300.

Walker SP, Chang SM, Powell CA. 2006. The association between early childhood stunting and weight status in late adolescence. Int J Obes 31:347-352.

Walker SP, Gaskin PS, Powell CA, Bennett FI. 2002. The effects of birth weight and postnatal linear growth retardation on body mass index, fatness and fat distribution in mid and late childhood. Public Health Nutr 5:391-396.

Way AB. 1976. Morbidity and postneonatal mortality. In: Baker PT, Little MA, editors. Man in the Andes: A Multidisciplinary Study of High Altitude Quechua. Stroudsburg, Pennsylvania: Dowden, Hutchinson and Ross, Inc. p 147-160.

Wells JCK. 2000. A Hattori chart analysis of body mass index in infants and children. Int J Obes Relat Metab Disord 24:325-9.

Wells JCK. 2010. Maternal capital and the metabolic ghetto: An evolutionary perspective on the transgenerational basis of health inequalities. Am J Hum Biol 22:1-17.

Wells JCK. 2012a. Ecogeographical associations between climate and human body composition: Analyses based on anthropometry and skinfolds. Am J Phys Anthropol 147:169-186.

Wells JCK. 2012b. Obesity as malnutrition: The role of capitalism in the obesity global epidemic. Am J Hum Biol 24:261-276.

Wells JCK, Chomtho S, Fewtrell MS. 2007. Programming of body composition by early growth and nutrition. Proc Nutr Soc 66:423-434.

Wells JCK, Cole TJ. 2011. Disentangling the size and adiposity components of obesity. Int J Obes 35:548-549.

Wells JCK, Cole TJ. In press. Height, adiposity and hormonal cardiovascular risk markers in childhood: how to partition the associations? Int J Obes.

Wells JCK, Cortina-Borja M. 2013. Different associations of subscapular and triceps skinfold thicknesses with pathogen load: An ecogeographical analysis. Am J Hum Biol 25:594-605.

Wells JCK, Stock JT. 2011. Re-examining heritability: genetics, life history and plasticity. Trends Endocrinol Metab 22:421-428.

WHO Expert Committee on Physical Status. 1995. Physical Status: The Use and Interpretation of Anthropometry. Geneva: World Health Organization.

WHO Multicentre Growth Reference Study Group. 2006. WHO Child Growth Standards: Length/height-for-age, weight-for-age, weight-for-length, weight-for-height and body mass index-for-age: Methods and development. Geneva: World Health Organization.

Wilson HJ, Dickinson F, Griffiths PL, Azcorra H, Bogin B, Varela-Silva MI. 2011. How useful is BMI in predicting adiposity indicators in a sample of Maya children and women with high levels of stunting? Am J Hum Biol 23:780-789.

Wilson HJ, Dickinson F, Hoffman DJ, Griffiths PL, Bogin B, Varela-Silva MI. 2012. Fat free mass explains the relationship between stunting and energy expenditure in urban Mexican Maya children. Ann Hum Biol 39:432-439.

Wren RE, Blume H, Mazariegos M, Solomons N, Alvarez JO, Goran MI. 1997. Body composition, resting metabolic rate, and energy requirements of short- and normal-stature, low-income Guatemalan children. Am J Clin Nutr 66:406-12.

**Figure Legends**

**Fig 1. Scatterplots of BMI z scores (a) and waist circumference-height ratio (WCHtR: b) against height and tibia length z scores.** Interaction for height or tibia length z score and site (highland or lowland) is highly significant (≤0.001 in all cases).

**Fig 2. Scatterplots of BMI z score against height z score demonstrating the impact of increasing measurement error in height on the stature-BMI relationship.**

**a. BMI**
