# Supplementary material for: Stunting, adiposity, and the individual-level “dual burden” among urban lowland and rural highland peruvian children
Source: Am J Hum Biol. 2014 Apr 7;26(4):481–90. doi: 10.1002/ajhb.22551 (PMC4312888; doi:10.1002/ajhb.22551)

Supplementary Information

Supplementary Table 1. Sample size and summary statistics by age and study site for height-, weight- and BMI-for-age z scores (WHO Multicentre Growth Reference Study Group 2006; de Onis and others 2007) and WCHtR.

Age (years)		Lowland				Highland			
		Height z score	Weight z score	BMI z score	WCHtR	Height z score	Weight z score	BMI z score	WCHtR
**0.5-1.99**	**Mean**	0.0	0.3	0.4	0.6	-1.9	-1.1	0.0	0.6
	**SD**[a]	1.02	0.9	0.84	0.04	0.78	0.6	0.85	0.02
	**n**	44	44	44	35	43	43	43	7
**2.0-8.5**	**Mean**	-0.4	0.4	0.8	0.5	-2.1	-1.3	0.2	0.5
	**SD**	0.81	1.05	1.07	0.05	0.96	0.84	0.7	0.04
	**n**	139	137	137	130	118	118	118	104
**9.5-14.5**	**Mean**	-0.6	0.8	1.0	0.5	-1.8	-1.1	-0.3	0.5
	**SD**	0.97	1.09	0.98	0.05	0.64	0.63	0.75	0.03
	**n**	60	15	60	60	37	12	37	30

[a] SD = standard deviation.
Sample sizes vary for individual measurements due to missing data points. Data are for combined sexes.

Supplementary Figures

Supplementary Figure 1. Map of Peru showing location of study communities

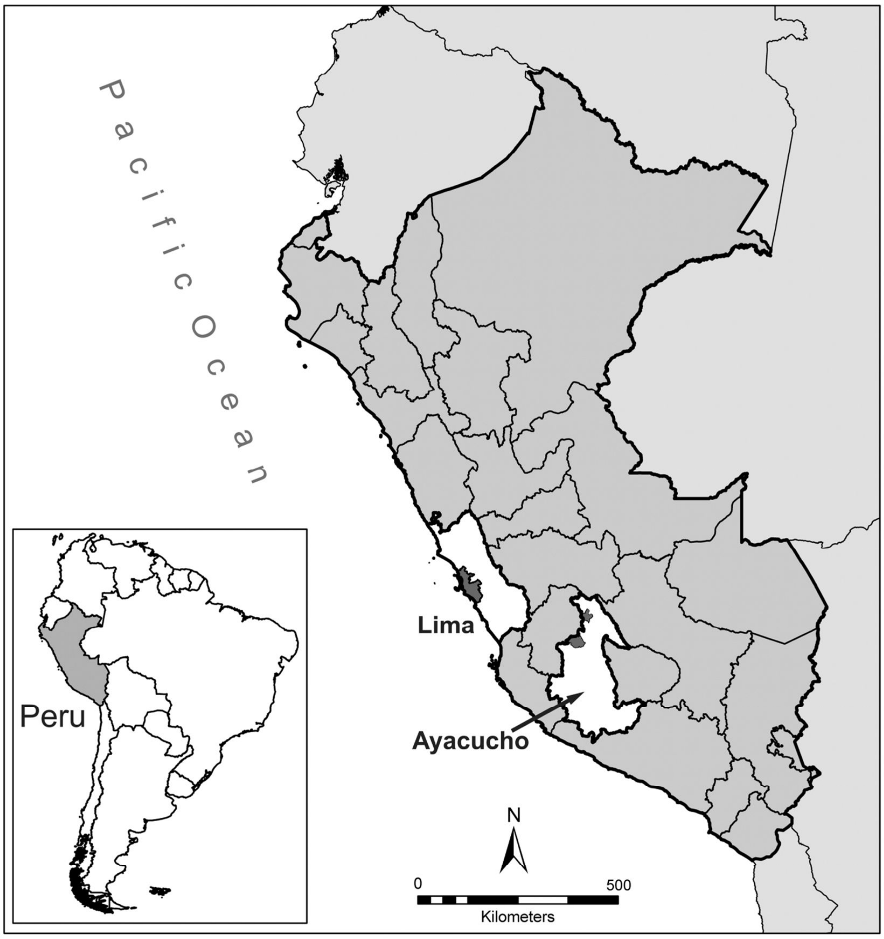

Supplementary Figure 2. Stunting rates by sample and age according to WHO criteria and growth standards/references

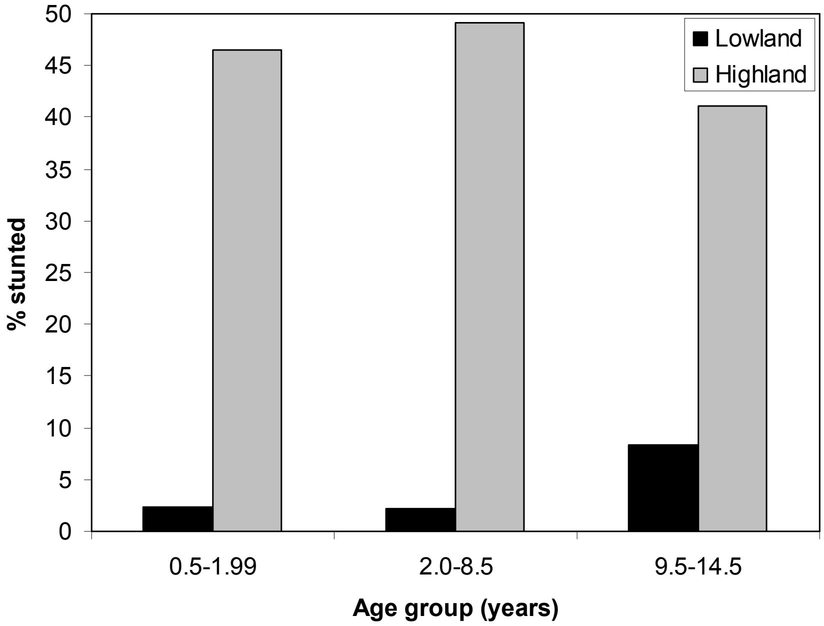

Supplementary Figure 3. Percentage of children in each IOTF BMI-for-age category from the age of 2 years in the lowland and highland samples

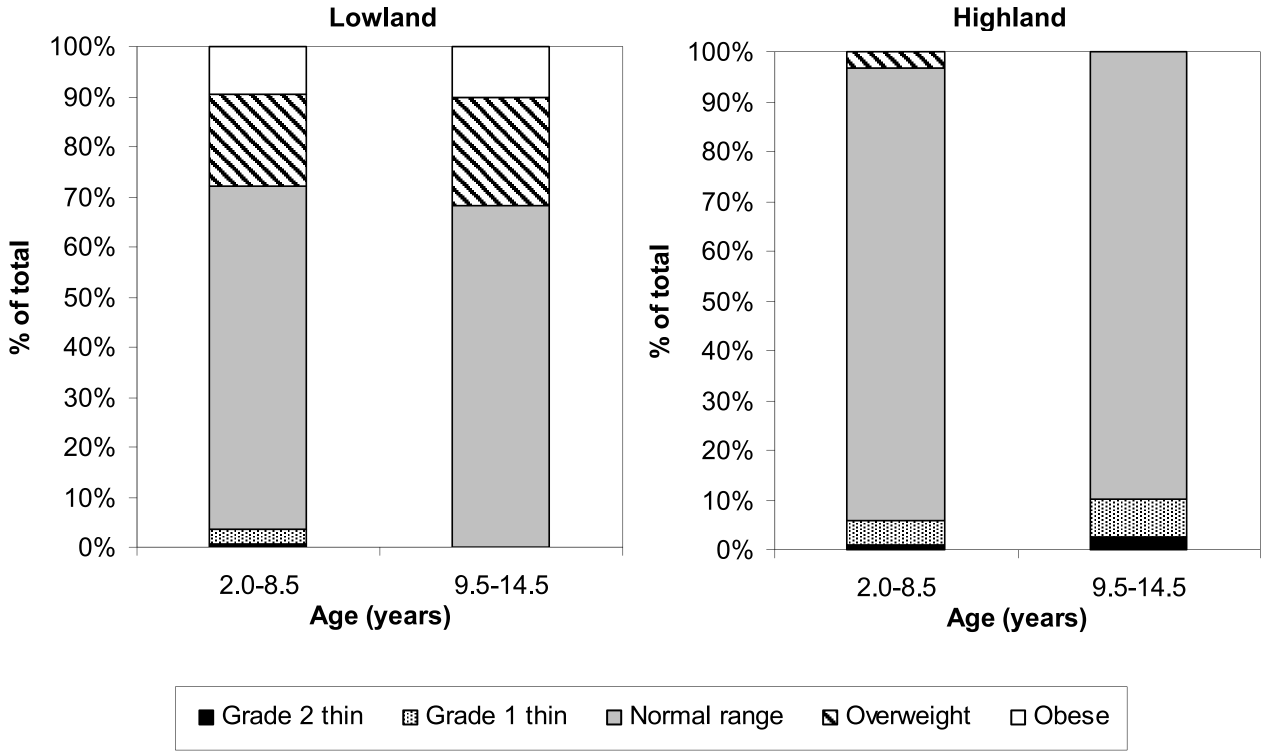

Supplementary Figure 4. Proportion of lowland and highland children with waist circumference: height ratio over 0.5 by age group

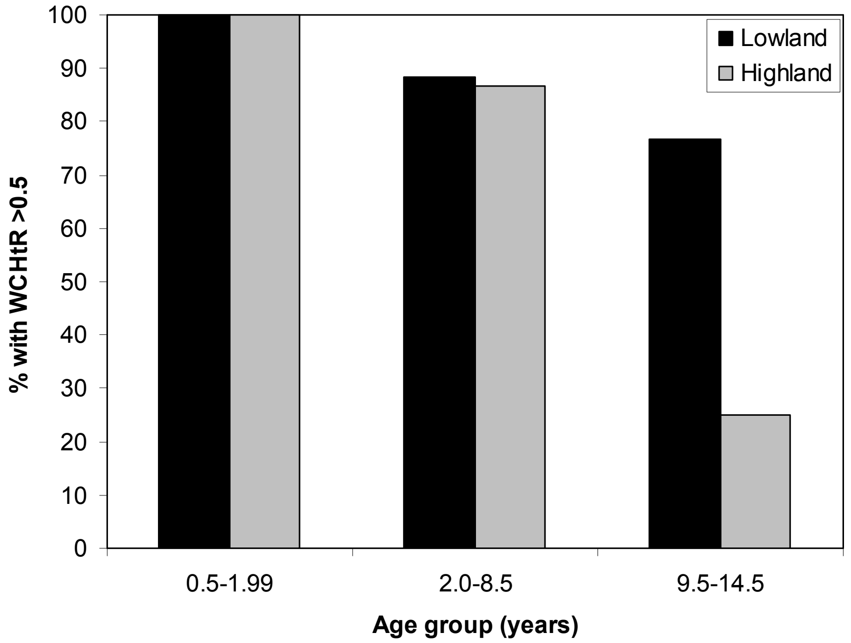

Supplement: Supplementary file 2 [file ajhb0026-0481-sd2.pdf]
